# Supplementary material for: CRISPR/Cas9-Mediated Deletion of Large Genomic Fragments in Soybean
Source: Int J Mol Sci. 2018 Dec 1;19(12):3835. doi: 10.3390/ijms19123835 (PMC6321276; doi:10.3390/ijms19123835)
Supplement: Supplementary file 1 [file ijms-19-03835-s001.pdf]

Table S1. Primer sequences used in the present study.

Primer Name	Primer Sequence	Purpose
Primer1	ATTCATAACAAAGCAAACGAG	To amplify the region spanning the target site *GmFT2a*-D1
Primer2	ACTTGACCTTCCCTTAAACAC	
Primer3	TTGATCCCGATGCACCTAGC	To amplify the region spanning the target site *GmFT2a*-D2
Primer4	CTAGCAAGCAAACATTGTGCG	
Primer5	TGAGCGTCAAAGTAGATCAAG	To amplify the region spanning the target site *GmFT2a*-D3
Primer6	CTTGCTGCTACCAACTGAGC	
Primer7	ATCGACCGATCGAGGACAAC	To amplify the region spanning the target site *GmFT5a*-D1
Primer8	TGGGAGACTACAGAAGCAAAGA	
Primer9	ACCACAAATGCAAGCTTTGGTAA	To amplify the region spanning the target site *GmFT5a*-D2
Primer10	TCAGCTGGAGTAAGGCATCCA	
Primer11	ACCCGTCAAGATCTCTTTATT	To detect large fragment deletions between the target sites *GmFT2a*-D1 and *GmFT2a*-D2
Primer12	ACGAGTTTAGGAATCAGTGTTCA	
Primer13	TCAAACACAATGGAATCGAGGC	To detect large fragment deletions between the target sites *GmFT2a*-D1 and *GmFT2a*-D3
Primer14	CACCCACACAAATCCGAAAGT	
Primer15	GGAGAAAAGAAAACATTTCATCG	To detect large fragment deletions between the target sites *GmFT5a*-D1 and *GmFT5a*-D2
Primer16	CTCTAAAGTATCAGCTGGAGTAAGG	
GmActin-F	CGGTGGTTCTATCTTGGCATC	To amplify *GmActin* as a normalization control.
GmActin-R	GTCTTTCGCTTCAATAACCCTA	
Cas9-F	TTGGGGCTCACACCAAACTT	To amplify part of the Cas9 coding sequence
Cas9-R	CGATCGCCTTCTTTTGCTCG	
